# The Automatic Classification of Pyriproxyfen-Affected Mosquito Ovaries

**DOI:** 10.3390/insects12121134

**Published:** 2021-12-17

**Authors:** Mark T. Fowler, Rosemary S. Lees, Josias Fagbohoun, Nancy S. Matowo, Corine Ngufor, Natacha Protopopoff, Angus Spiers

**Affiliations:** 1 Department of Vector Biology, Liverpool School of Tropical Medicine, Liverpool L3 5QA, UK; rosemary.lees@lstmed.ac.uk (R.S.L.); angus.spiers@innovation2impact.org (A.S.); 2 Centre de Recherche Entomologique de Cotonou (CREC), Cotonou BP 2604, Benin; jfagbohoun@crec-lshtm.org; 3 Faculty of Infectious and Tropical Diseases, London School of Hygiene & Tropical Medicine, London WC1E 7HT, UK; Nancy.Matowo@lshtm.ac.uk (N.S.M.); corine.ngufor@lshtm.ac.uk (C.N.); Natacha.Protopopoff@lshtm.ac.uk (N.P.); 4 Mwanza Medical Research Centre, Department of Parasitology, National Institute for Medical Research, Mwanza P.O. Box 1462, Tanzania

**Keywords:** *Anopheles* mosquito, fertility, ovary development, pyriproxyfen (PPF), side-effects, machine learning, image classification, automated identification, convolutional neural network

## Abstract

**Simple Summary:**

With resistance to the pyrethroid family of insecticides increasing, it is more important than ever that tools are available to measure the efficacy of alternatives. Pyriproxyfen (PPF) is an alternative insecticide whose mode of action sterilises adult mosquitoes. Consequently, the efficacy of PPF-based tools can be measured through visual examination of egg development by trained experts. This, however, can be a time-consuming process for which the required expertise can be difficult to train and is not available in many contexts. As such, we propose that an objective machine learning program, which can automatically classify the fertility status of adult mosquito ovaries via a colour image, be developed to improve the speed, accuracy, and consistency of assessment. This study shows that a convolutional neural network, built in Python’s TensorFlow library, can quickly classify images of dissected ovaries into either ‘fertile’ or ‘infertile’ with a high accuracy rate. Such an application would be a practical and accessible tool available to all researchers studying the efficacy of PPF or other insecticides with a similar mode of action.

**Abstract:**

Pyriproxyfen (PPF) may become an alternative insecticide for areas where pyrethroid-resistant vectors are prevalent. The efficacy of PPF can be assessed through the dissection and assessment of vector ovaries. However, this reliance on expertise is subject to limitations. We show here that these limitations can be overcome using a convolutional neural network (CNN) to automate the classification of egg development and thus fertility status. Using TensorFlow, a resnet-50 CNN was pretrained with the ImageNet dataset. This CNN architecture was then retrained using a novel dataset of 524 dissected ovary images from *An. gambiae* s.l. *An. gambiae* Akron, and *An. funestus* s.l., whose fertility status and PPF exposure were known. Data augmentation increased the training set to 6973 images. A test set of 157 images was used to measure accuracy. This CNN model achieved an accuracy score of 94%, and application took a mean time of 38.5 s. Such a CNN can achieve an acceptable level of precision in a quick, robust format and can be distributed in a practical, accessible, and free manner. Furthermore, this approach is useful for measuring the efficacy and durability of PPF treated bednets, and it is applicable to any PPF-treated tool or similarly acting insecticide.

## 1. Introduction

Insecticide-treated nets (ITNs) are a common vector control tool and have considerably decreased the burden inflicted by malaria [1]. However, in recent years, species of the mosquito genus *Anopheles*, the principal vector for malaria, have demonstrated an increased resistance to the pyrethroid-based insecticides used to treat ITNs. This increase in resistance to pyrethroids threatens the efficacy of ITNs and may have contributed to an increase in malaria cases in affected areas [2]. Consequently, alternative effective insecticides for use on ITNs need to be identified to maintain the efficacy of this intervention and meet the gap in global disease control that pyrethroid resistance has created [3,4]. ITNs treated with a mixture of pyriproxyfen (PPF) and pyrethroids offer an alternative to standard pyrethroid-treated ITNs in areas where pyrethroid-resistant malaria vectors are prevalent [5,6,7,8]. The mode of action of PPF affects the fertility, longevity, and lifetime fecundity of malaria vectors [9,10], and PPF-treated ITNs have been shown to sterilise *Anopheles* mosquitos under both laboratory and field conditions [11,12]. As vector ovary development is inhibited by exposure to PPF [8], and females that fail to develop morphologically normal eggs have been shown to not oviposit [13,14], a means of measuring efficacy and monitoring the durability of PPF and PPF-treated tools is through the assessment of eggs for signs of abnormal or inhibited development [8,12]. Although different means of scoring sterility exist (e.g., by looking for the ability to prevent egg laying or oviposition inhibition), another method to determine fertility status is based on trained experts manually dissecting ovaries and classifying egg development according to Christopher’s stages [15]. However, this can be a time-consuming process and requires a level of expertise not always available. Therefore, to increase the throughput and robustness of data used to measure the efficacy and durability of PPF-based ITNs, and to aid efficient and reproducible data collection in research settings, freely available alternative methods for the accurate, quick, and automatic classification of ovary development are required.

In recent years, deep learning models and convolutional neural networks (CNNs) have made significant progress across a range of computer vision problems, including image classification [16]. A CNN implements a convolution operation across several distinct layers to convert an input (i.e., an image) into an output (i.e., a classification)**.** The convolution operation applies a filter or kernel (usually a 3 × 3 or 5 × 5 matrix) to a two-dimensional representation of an image. This matrix then slides over the full 2D grid, performing calculations on the data depending on the kernel’s weights, transforming data into a representation of patterns found within the image (i.e., edges, etc.) [17]. A CNN, therefore, uses linear regression with forward and backward propagation in a neural network to automatically adjust and determine the most appropriate kernel weights [18,19]. These weights can then identify different pattern types found within a dataset, with layers earlier in the network identifying primitive features in an image, such as edges and colours, while deeper layers detect more complex shapes, patterns, or objects [20,21].

This type of architecture enables the automatic training and detection of multiple visual features, which can then be used to identify and classify variance between images. However, the area of application for deep learning and CNNs has been constrained by its reliance on large datasets to avoid overfitting (i.e., to ensure generalisability) and, thus, achieve high accuracy rates [22]. Nevertheless, the size of a dataset can be increased through data augmentation, which employs a raft of tactics so as to artificially increase the available dataspace and allow generalisable models to be built. Data augmentation includes the geometric transformation, colour augmentation, and random cropping of available data (amongst other techniques), thereby creating randomised novel images from those that are already available [23]. However, even with data augmentation, most datasets are still insufficient to avoid overfitting. In such cases, transfer learning can be used, whereby opensource architectures and pretrained weights, derived using very large datasets, are repurposed and fine-tuned for a different but related task [24]. Models trained against the ImageNet dataset (which contains over 14 million images and 20 thousand classes) are freely available and regularly achieve high levels of accuracy [25]. Three common and high-performing models used in transfer learning, all pretrained and tested against the ImageNet dataset, are (1) VGG-16 [26], (2) ResNet-50 [27], and (3) InceptionV3 [28,29].

Machine learning has already been successfully utilised within entomology for a number of species classification tasks, such as the identification of pest insect species [30], the recognition of lepidopteran species [31], and the classification of mosquito species [32,33,34,35]. Additionally, automatic tools have been developed to count the eggs laid by female mosquitos, which can be used to estimate fecundity [36,37,38]. However, current work on the automatic classification of mosquito fertility and egg development is limited. As such, this study is aimed at bridging this gap and uses deep learning, data augmentation, and transfer learning to develop a quick, robust, and practical method to classify the fertility status (i.e., ‘fertile’ or ‘infertile’) of mosquito ovaries from colour images. To be successful, this new method must (1) be automatic and require no, or limited, expert knowledge to categorise an image, (2) achieve close to the human accuracy rate of 99–100% (rate determined by the agreement between two scorers assessing the dataset used in this study), (3) be in an easily distributable, non-proprietary, and low-cost format, and (4) classify ovary fertility of an image faster than the estimated 2 s taken by human experts (rate determined by the mean time taken for four trained technicians to classify 30 random ovary images).

Using a novel dataset of dissected ovary images, data augmentation, and transfer learning, we were able to build and train a CNN in TensorFlow that can detect and classify the development status (‘fertile’ or ‘infertile’) of 157 ovaries in 38.5 s at a 94% accuracy rate. As such, this study proposes a new method for the automatic classification of the fertility status of *Anopheles* mosquito ovaries that is quick, accurate, and easily distributable, and that is not dependent on trained experts to score egg development.

## 2. Materials and Methods

### 2.1. Image Dataset

As no publicly available datasets exist, data from ongoing research were used for this study. A total of 524 images of dissected ovaries from 5–8 day old female mosquitos were collected and labelled with the appropriate fertility status (based on Christopher’s stage of egg development). These were all full colour images obtained from three sources where fertility status was determined and corroborated by two trained expert scorers. A summary of the datasets used here is found in Table 1.

**Figure 1:**
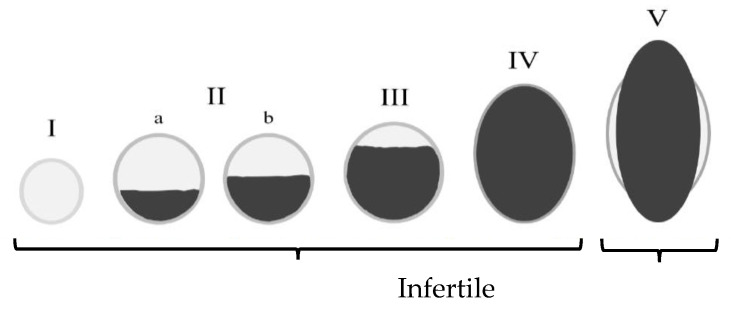
Christopher stages of egg development. Mosquitos whose eggs have fully developed to stage V (normal elongated, boat/sausage-shaped eggs with lateral floats) are classified as ‘fecund’ or ‘fertile’. If eggs have not fully developed and remain in stages I–IV (less elongated, round shape, lacking floats), the mosquito is classified as ‘non-fecund’ or ‘infertile’.

The first dataset contained a total of 124 blood-fed adult pyrethroid-resistant female *An. gambiae* s.l. mosquitoes which had survived exposure to either a control untreated net or a PPF-treated net (Royal Guard) in experimental hut studies performed in accordance with current WHO guidelines [39]. Mosquitoes were collected as wild free-flying adults in experimental huts in Cove, Southern Benin, with 36.3% (*n* = 45) classified as being infertile and the remaining 63.7% (*n* = 79) classified as being fertile. The second dataset contained 187 blood-fed adult pyrethroid-resistant female *An. gambiae* Akron mosquitoes from insectary-maintained colonies. All samples had survived exposure to either a control untreated net or a PPF-treated net (Royal Guard) in WHO cone bioassays [39]. Of the total samples in the second dataset, 77.0% (*n* = 144) were classified as being infertile, and 23.0% (*n* = 43) were classified as being fertile. All samples in the first and second dataset were, after exposure, held in plastic holding cups and provided 10% glucose for 72 h to allow enough time to become gravid. Prior to dissection, mosquitoes were killed by placing them in a freezer at −20 °C for 5–10 min and then dissected on a dissecting slide by separating the abdomen from the head and thorax to expose the ovaries using dissecting needles. After dissection, eggs and ovaries of each mosquito were observed and photographed using a microscope equipped with a digital camera at 4× or 10× magnification. Developmental status of the eggs in each mosquito’s ovaries was classified and validated by two scorers according to Christopher’s stage of egg development [15]. Mosquitoes were classified as ‘fertile’ if eggs had fully developed to Christopher stage V and ‘infertile’ if eggs had not fully developed and remained in stages I–IV (see Figure 1).

The third dataset contained 125 free-flying freshly blood-fed pyrethroid-resistant female *An. funestus* s.l. mosquitoes collected from the wall and roof of houses in Mwanza, Northwest Tanzania. Of these mosquitos, 46.4% (*n* = 58) were classed as being infertile and 53.6% (*n* = 67) were classified as being fertile. Dataset 4 also contained free-flying freshly blood-fed pyrethroid-resistant female mosquitoes collected from the wall and roof of houses in Mwanza, Northwest Tanzania. However, these were *An. gambiae* s.l., 56.8% (*n* = 50) classed as infertile and 43.2% (*n* = 38) classed as fertile. All samples from datasets 3 and 4 were, after collection and following the CDC bottle bioassay guidelines [40], immediately exposed to glass bottles treated with 1× the diagnostic dose of 100 μg/mL of PPF solution or control bottles treated with acetone for 60 min and left for 72 h post exposure to allow time to become gravid. Dissection was then carried out under a stereoscopic dissecting microscope (using a Nikon MODEL C-PSN) at 5× magnification to assess ovary development. The status of ovaries and eggs was again categorised by two scorers as either ‘fertile’ or ‘infertile’ according to Christopher’s stage of egg development, with those in Christopher stage V determined to be ‘fertile’ and those in stages I–IV classed as ‘infertile’ [13]. After dissection, one image per mosquito was captured with a Motic camera microscope into a tablet PC.

### 2.2. Pre-Processing and Train/Test Split

**Figure 2:**
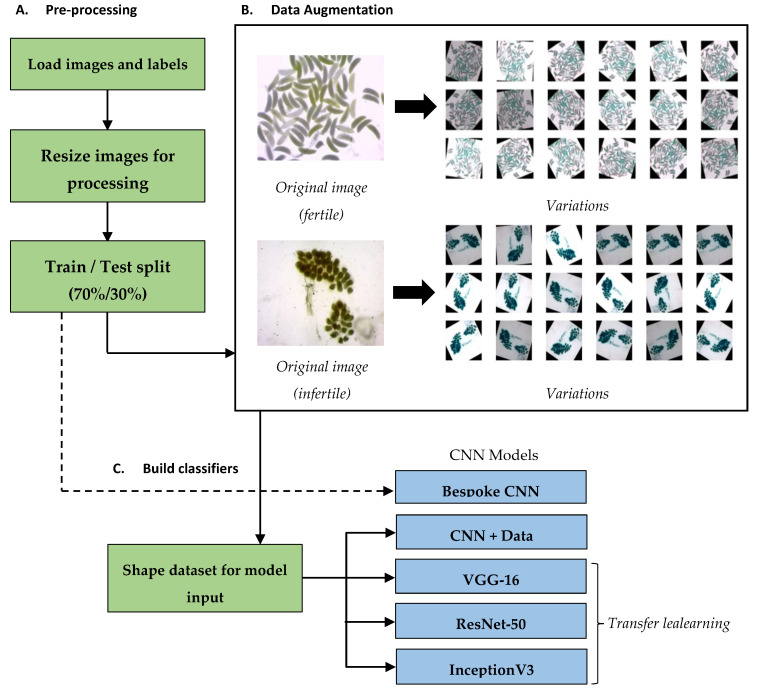
Summary of analysis workflow. (**A**) Data are pre-processed as described in Section 2.2. Images and labels are loaded before the images are resized and undergo a random 70%/30% split into training and test sets. (**B**) The training set undergoes data augmentation as described in Section 2.3. Each original image produces 18 variations based on a random rotation around 360°, a random horizontal flip, a random vertical flip, and a random brightness shift between 60% and 140%. Each variation retains the same classification as its original. (**C**) The training set is fitted to a range of CNNs, and classifiers are built and tested as described in Section 2.4.

After data were loaded into Python, all images were rescaled to 224 × 224 pixels to ensure consistency and improve processing times (Figure 2A). Before data were analysed, images were first randomly allocated to a training and a test set using a respective split of 70% (*n* = 367) and 30% (*n* = 157). A training set is used to teach a model to classify the correct domain. The set used here to train the model consisted of a total of 367 images, 151 (41.1%) classed as fertile and 216 (58.9%) classed as infertile. The test set is used to measure the accuracy of a model. Here, 157 total images were allocated to testing accuracy, with 76 (48.4%) classed as fertile and 81 (51.6%) classed as infertile.

### 2.3. Data Augmentation 

To overcome the limited dataset, data augmentation was employed to increase the number of images available for training. A total of 18 random transformations were applied to each image in the training set. The original images were retained, and each variant maintained its original’s classification label (‘fertile’ or ‘infertile’). Each variant underwent a random transformation along four dimensions: (1) a random rotation around 360°; (2) a randomised horizontal flip; (3) a randomised vertical flip; (4) a random brightness shift between 0.6 and 1.4. See Figure 2B for examples of this data augmentation on a fertile and an infertile ovary image. When images are rotated, a void is created around the edges. These voids can be filled by a number of means (e.g., by repeating the whole image or the neighbouring pixel). Experimentation found that leaving these voids black had the least impact on classification. Data augmentation was only applied to the training set, increasing it from 367 to 6973 images. The test set did not undergo any data augmentation.

### 2.4. Analysis

To prepare data for processing by the CNNs, all images were resized (Figure 2C). The dimensions of each image were rescaled to the correct input shape for the training algorithm (224 × 224 pixels for the bespoke CNN, VGG-16, and ResNet-50 and 229 × 229 for InceptionV3). Resizing images in this manner also ensures that the magnification, resolution, or quality of the photos available when using the tool do not affect classification.

Before transfer learning was undertaken, a benchmark was established using a bespoke handmade CNN in TensorFlow. The architecture used for this CNN comprised a ReLU activated 3 × 3 input layer with 16 nodes, a (1, 1) stride, and ‘same’ padding (so that output size was equal to input size). This input layer then fed into three 3 × 3 ReLU activated hidden layers, with the same stride and padding as the input layer and whose number of nodes doubled from the previous layer (e.g., 16, 32, 64, and 128). Each convolutional layer fed into a 2 × 2 pooling layer, with a (2, 2) stride, to prevent overfitting. The final hidden layer was used as the input into a binary densely connected softmax output layer to capture either fertile (0) or infertile (1). As the model was a binary classifier, it was compiled using the ‘Sparse Categorical Cross-Entropy’ cost method, ‘Root-Mean-Squared Propagation’ optimiser, and ‘Binary Accuracy’ metric [41]. The model was trained against two training sets and used to generate two classifiers. The first classifier was trained against the original, pre-data augmentation, training set (i.e., 367 images) and the second full training set including data augmentation (i.e., 6973 images). During fitting, experimentation found that five epochs and a batch size of 32 produced the optimal performance. These models provided two benchmarks showing the impact of both data augmentation and transfer learning.

Once a benchmark was established, transfer learning was undertaken. The VGG-16 [26], ResNet-50 [27], and InceptionV3 [29] architectures with parameters pretrained against the ImageNet dataset were repurposed using the full training set (i.e., 6973 images). Although the architectures’ layers were frozen, to maintain their ImageNet weighting, each was slightly altered for its new purpose. The output layer of each architecture was replaced with a densely connected softmax layer with two outputs, so as to accommodate the binary classification of ‘fertile’ or ‘infertile’. These altered models were then compiled and fit to the training set. As each model is a deep net classifying a binary problem, all three were compiled using the ‘Sparse Categorical Cross-Entropy’ cost method, ‘adam’ optimiser, and ‘Binary Accuracy’ metric [41]. The training data were then used to improve the target predictive function of the architectures to detect and classify fertility status. When fitting, manual fine-tuning of the models’ hyperparameters found that five epochs and a batch size of 32 maximised performance.

### 2.5. Resources and Requirements 

Image pre-processing, data augmentation, and analysis were performed using the TensorFlow 2.4.1 library in Python through a Jupyter notebook created for this project by the lead author. All analysis found here was performed on an Intel 2.20 GHz 10 Core Xeon Silver 4114 CPU equipped on a desktop computer with 25.8 GB of RAM.

## 3. Results

For this study, one bespoke CNN architecture was created to classify data, and transfer learning was used to repurpose the existing VGG-16, ResNet-50, and InceptionV3 architectures. All architectures except one were retrained using the augmented training set (e.g., 6973 images). A version of the bespoke CNN was trained against the original, pre-data augmentation training set (e.g., 367 images) for benchmarking. The accuracy of all models was then measured against the test set (157 images). For a summary of the model performance, see Table 2.

### 3.1. Classification Accuracy

Accuracy was measured by comparing a model’s classification of the images within the test set against that of the human experts, with a final accuracy rate calculated by dividing the number of correct predictions of ‘fertile’ or ‘infertile’ by the number of total predictions. Recall and precision were measured to ensure there was no imbalance in the accuracy of classes. The bespoke architecture achieved a benchmark of 78% without data augmentation but had significant skew toward ‘fertile’ predictions. Accuracy increased to 82%, with less skew toward ‘fertile’, when the augmented training set was used. When transfer learning was employed, the VGG-16 architecture achieved an accuracy of 89% with a satisfactory balance between classification, and the InceptionV3 architecture reached an accuracy similar to the benchmark of 80%, but with slight skew toward ‘infertile.’ However, the ResNet-50 architecture was able to attain the highest performance of all architectures, scoring an accuracy of 94% with a good balance between classes when measured against the test set. This is a level of precision close to the human accuracy rate of 99%. For a confusion matrix detailing the ResNet-50 architecture’s performance, in this instance, see Table 3. As the images used to train and test the were all pre-processed, the magnification, resolution, or quality of image should not affect classification, and the accuracy scores reported here should be representative of real-world use.

### 3.2. Classification Speed

All models were able to classify the full test set in under 1 min. To import all the necessary Python libraries, build the architecture, load the architecture with the pretrained fertility classification weights, and get the model’s fertility prediction for the 157 images in the test set took a mean time (over five repetitions) of 28 s for the bespoke architecture, 41.7 s using the VGG-16 architecture, 38.5 s for ResNet-50, and 36.5 s for InceptionV3. This compares with an estimated 5 min 14 s taken for one human expert to classify the same number of images (figure determined by multiplying 157 by the mean time of 2 s taken for four trained technicians to classify 30 random ovary images).

## 4. Discussion

This study aimed to use deep learning, data augmentation, and transfer learning to develop an automatic method for the classification of mosquito fecundity. It was determined that, for a solution to this problem to be appropriate, it must (1) require no, or limited, expert knowledge to categorise an image, (2) achieve close to the human accuracy rate of 99–100%, (3) be in an easily distributable, non-proprietary, and low-cost format, and (4) classify an image faster than the estimated 2 s taken by human experts.

As such, we propose that a ResNet-50 CNN architecture [27], trained against the ImageNet database, be repurposed and fine-tuned to classify the fertility status (‘fertile’ or ‘infertile’) of *Anopheles* mosquito ovaries. Classification was based on Christopher’s stages of egg development [15], with eggs in stage V classed as ‘fertile’ and those eggs remaining in stages I–IV labelled as ‘infertile.’ Here, we show that such a model is capable of automatically classifying 157 images with a 94% accuracy rate in less than 40 s. Furthermore, as the model is built using TensorFlow 2.4.1, it uses a freely available, accessible, and robust opensource technology that is easily distributable via the web or mobile phones [41]. Consequently, the approach detailed in this study meets three of its aims, as it does not require any experts to categorise an image, it is easily distributable in a free format, and it can classify images faster than an expert. However, although the accuracy rate of the model does not achieve that of a human expert, it is still highly precise and is only 5% less accurate than trained experts. Furthermore, it is likely that this accuracy rate of 94% can be raised as more data become available.

Such a model is useful when assessing the efficacy of PPF-based tools through measurements of induced sterility in laboratory reared and field-collected populations of mosquitoes [12], and it could be particularly useful for large-volume bioassays done for durability monitoring of bio-efficacy of PPF-treated ITNs distributed in disease-endemic communities over time. It can also be used in bioassays performed during resistance monitoring, whereby field-collected females are exposed to a discriminating concentration of PPF to measure induced sterility [8]. This is a practical and accessible tool available to all researchers studying the efficacy of PPF or other insecticides with a similar mode of action.

Although offering several advancements over the existing manual method for classifying ovary status via dissection and examination, the model presented here is subject to its own limitations. Machine learning will not remove the need for trained technicians to dissect ovaries, only the assessment of their fertility status. Consequently, some equipment and expertise to dissect samples and to take digital colour images are still required to use the model. However, as taking photos of dissected ovaries is standard practice for record keeping and quality control, this model’s need for images should not add additional work but increase objectivity and reproducibility while removing the need for a second trained technician to confirm classification. A second limitation to the current model comes from the dataset included in its training. As only pyrethroid-resistant *Anopheles* mosquito ovaries exposed to PPF were included in this study, its results are not generalisable to other species, arthropods, or insecticides. Thirdly, as there are no established dissection and imaging guidelines for capturing mosquito ovaries, there may be considerable divergence between the methods and tools employed at different sites. This may mean that the model is currently only generalisable to those locations that use techniques similar to those detailed in this paper’s methods. However, the scale of this divergence, if any, is not currently known. Lastly, although a distributable application of the ResNet-50 model is currently in development, a version of the tool accessible via the internet is not yet available. Consequently, some knowledge of Python is currently necessary to employ the classifier.

It is likely that developments can be made to improve performance and accessibility. For example, to increase accuracy and applicability of the classification tool, the training set could be expanded to include samples exposed to other growth regulators or insecticides of interest, images from a broader range of sites, or other species of mosquito (including all cryptic subspecies of the *An. gambiae* complex). Additionally, accuracy and generalisability may be increased through the use of a fuzzy image classifier or classification using fuzzy logic, rather than a CNN. This alternative approach may improve precision as it could account for any ambiguity in the image dataset [42]. Furthermore, as the current model is limited to a binary classification of ‘fertile’ or ‘infertile’, it could be developed to capture the five Christopher stages of egg development or count the number of eggs in the dissected ovaries. Moreover, although the use of images from multiple locations in this study should ensure that the model is robust enough to deal with differences in the dissection and imaging of samples, standard operating procedures concerning dissection and imaging need be developed to support the use of the classification tool. Lastly, the ResNet-50 model is currently only available via a Jupyter notebook; however, as a version of the model that can be accessed via the web is in development, a free and easy-to-use version of the model could be made freely available.

## 5. Conclusions

In conclusion, the reliance on manual scoring of mosquito egg development to determine the impact of PPF on the fertility of *Anopheles* mosquitos requires a level of expertise and experience that is not always available. This paper shows these limitations can be overcome using a ResNet-50 CNN model that automates the classification of egg development as a measure of fertility status. Such a model is fast, can achieve an acceptable level of precision, is in a robust format, and has the potential to be easily distributed in a practical, accessible, and freely available manner. Furthermore, this approach is applicable to scoring the fertility of mosquitoes exposed to any PPF-treated tool or similarly acting insecticide or insect growth regulator, which causes the same impact on ovary morphology, as well as applicable during bioassays performed to measure efficacy of these tools and in resistance monitoring.

## Figures and Tables

**Table 1:** Image dataset summary.

Dataset	Source	Strain	Image Count	Fertile	Infertile
1	Cove, Benin	*An. gambiae* s.l.	124	79	45
2	Insectary colony	*An. gambiae* Akron	187	43	144
3	Mwanza, Tanzania	*An. funestus* s.l.	125	67	58
4	Mwanza, Tanzania	*An. gambiae* s.l.	88	38	50
Total			524	227	297

**Table 2:** Performance of transfer learning architectures against the test set.

Architecture	Accuracy ^1^	Recall (Fer) ^2^	Recall (Inf) ^3^	Precision (Fer) ^4^	Precision (Inf) ^5^	Speed ^6^
Bespoke CNN	0.777	0.951	0.592	0.918	0.713	28.1 s
CNN + data Augmentation	0.815	0.901	0.724	0.873	0.777	28.7 s
VGG-16	0.885	0.901	0.868	0.892	0.880	41.7 s
ResNet-50	0.943	0.951	0.934	0.947	0.939	38.5 s
InceptionV3	0.803	0.716	0.895	0.747	0.879	36.5 s

^1^ Accuracy—correct predictions divided by total number of predictions; ^2^ Recall (Fer)—fraction of fertile observations successfully retrieved; ^3^ Recall (Inf)—fraction of infertile observations successfully retrieved; ^4^ Precision (Fer)—true fertile predictions divided by total fertile predictions; ^5^ Precision (Inf)—true infertile predictions divided by total infertile predictions; ^6^ Speed—mean time (in seconds) over five repetitions for the model to load and classify 157 images.

**Table 3:** Confusion matrix for ResNet-50.

*n* = 157 ^1^	Predicted Infertile	Predicted Fertile	
**Actual Infertile**	77	4	81
**Actual Fertile**	5	71	76
	82	75	

^1^ Confusion matrix—comparison of actual values with those predicted by the model and giving details on true positive (top-left), true negative (bottom-right), false positive (top-right), and false negative (bottom-left) rates.

## Data Availability

All code and model weights used for analysis, modelling, and visualisation are available in a GitLab repository at https://gitlab.com/MTFowler/lstm_ovaryclassify (last accessed on 3 December2021). As the images in this study are still being used in ongoing research, they will only be available upon reasonable request.
